# Knowledge of and Attitudes to Influenza Vaccination in Healthy Primary Healthcare Workers in Spain, 2011-2012

**DOI:** 10.1371/journal.pone.0081200

**Published:** 2013-11-18

**Authors:** Angela Domínguez, Pere Godoy, Jesús Castilla, Núria Soldevila, Diana Toledo, Jenaro Astray, José María Mayoral, Sonia Tamames, Susana García-Gutiérrez, Fernando González-Candelas, Vicente Martín, José Díaz, Nuria Torner, the CIBERESP Working Group for the Survey on Influenza Vaccination in Primary Health Care Workers

**Affiliations:** 1 Departamento de Salud Pública, Universidad de Barcelona, Barcelona, Spain; 2 CIBER Epidemiología y Salud Pública (CIBERESP), Barcelona, Spain; 3 Agencia de Salud Pública de Cataluña, Barcelona, Spain; 4 Instituto de Salud Pública de Navarra, Pamplona, Spain; 5 Servicio de Epidemiología, Agencia de Salud Pública de Barcelona, Barcelona, Spain; 6 Área de Epidemiología, Comunidad de Madrid, Madrid, Spain; 7 Servicio de Vigilancia de Andalucía, Sevilla, Spain; 8 Dirección General de Salud Pública, Investigación, Desarrollo e Innovación, Junta de Castilla y León, León, Spain; 9 Hospital Galdakao-Usansolo, Unidad de Investigación, REDISSEC, Bizkaia, Spain; 10 Centro Superior de Investigación en Salud Pública, Universidad de Valencia, Valencia, Spain; 11 Instituto de Biomedicina, Universidad de León, León, Spain; 12 Servicio Andaluz de Salud, Sevilla, Spain; Fondazione IRCCS Ca' Granda Ospedale Maggiore Policlinico, Università degli Studi di Milano, Italy

## Abstract

Annual influenza vaccination is recommended for healthcare workers, but many do not follow the recommendation. The objective of this study was to investigate the factors associated with seasonal influenza vaccination in the 2011–2012 season. We carried out an anonymous web survey of Spanish primary healthcare workers in 2012. Information on vaccination, and knowledge and attitudes about the influenza vaccine was collected. Workers with medical conditions that contraindicated vaccination and those with high risk conditions were excluded. Multivariate analysis was performed using unconditional logistic regression. We included 1,749 workers. The overall vaccination coverage was 50.7% and was higher in workers aged ≥ 55 years (55.7%), males (57.4%) and paediatricians (63.1%). Factors associated with vaccination were concern about infection at work (aOR 4.93; 95% CI 3.72–6.53), considering that vaccination of heathcare workers is important (aOR 2.62; 95%CI 1.83–3.75) and that vaccination is effective in preventing influenza and its complications (aOR 2.40; 95% CI 1.56–3.67). No association was found between vaccination and knowledge of influenza or the vaccine characteristics. Educational programs should aim to remove the misconceptions and attitudes that limit compliance with recommendations about influenza vaccination in primary healthcare workers rather than only increasing knowledge about influenza and the characteristics of the vaccine.

## Introduction

Influenza is a highly contagious disease that causes a significant burden of morbidity and mortality in the community [1]. During the 2010-2011 season, the estimated overall rate of hospitalization for severe confirmed influenza in Spain was 5.76 cases per 100,000, although this probably underestimates the problem [2]. Healthcare workers are exposed to patients with influenza in the workplace and, consequently, are at risk of acquiring the disease and may act as vectors of nosocomial transmission. Therefore, vaccination is an essential element of prevention programs [3].

There are few Spanish studies of the relationship between knowledge, risk perception and the need for influenza vaccination in healthcare workers. Studies made before the emergence of the pandemic virus show vaccination coverages in healthcare workers of around 20% or less [4-7], much lower than the estimated coverage in the United States of between 62% and 76.6% [8]. Studies in various countries [9-11] and Spain [12-14] show evidence of behavioural changes with respect to influenza virus A (H1N1) pdm09 vaccination in hospital-based healthcare workers, but there are few studies in primary healthcare (PHC) workers.

Barriers to influenza vaccination of healthcare workers include misconceptions or lack of knowledge about influenza infection, the potential severity of the disease and the perception that the vaccine is not very effective [15-20]. Factors favouring vaccination include previous vaccination, the desire to protect oneself and one’s patients, and the perceived effectiveness of the measure [21-24].

To improve the appropriate use of vaccination as a preventive measure, in-depth knowledge of the issues related to the acceptance of influenza vaccination by PHC workers, who are the main facilitators and recommenders of vaccination to patients [25,26], is essential. The aim of this study was to investigate the association between influenza vaccination of PHC workers and knowledge of and attitudes to influenza vaccination and disease in Spain.

## Methods

A cross-sectional study was made by administering a questionnaire to PHC workers in 7 Spanish regions (Andalusia, Castile-Leon, Catalonia, Valencia, Madrid, Navarra and Basque Country), which represent 70% of the Spanish population. The questionnaire was conducted anonymously between March 1 and May 25, 2012 via the internet.

### Study subjects

The target population was any PHC worker providing direct patient care (family physicians, paediatricians and nurses). 

A list of PHC centres was obtained from each participating region. Thirty PHC centres were selected by simple randomized sampling. An email message was sent to all PHC workers from the selected centres which explained the study, invited the worker to participate and provided a link in order to complete the web-based survey. 

The questionnaire was accessible for a month and an email reminder was sent every 10 days to workers who had not accessed the questionnaire.

### Sample size

The simple size necessary to reach the study objectives was estimated considering a bilateral alpha error of 0.05, a statistical power of 0.8, a prevalence of the behaviours considered of 0.7 and an odds ratio of the prevalence of the behaviours considered in vaccinated subjects compared with unvaccinated subjects of 2.0. This showed that a minimum of 185 workers would need to be surveyed. As we planned to analyse the study objectives by strata according to age groups, years of professional work, and type of profession, the minimum number of surveys required was estimated at 925. 

Given that reports [10,27] suggest that the proportion of responses to surveys sent be email and answered on the internet is round 30%, it was considered necessary to send out a minimum of 3083 surveys.

### Variables

The questionnaire was developed after reviewing the scientific literature on the subject, especially the questionnaire used in the study by Kraut et al [10]. The questions were adapted to the specific circumstances of the Spanish National Health System and was tested on three occasions in a group of 20 healthcare workers. On the first two occasions, the survey was administered on paper in order to identify questions that might have been confusing and determine the response time required (mean 9.75 minutes; between 4.5 and 18.5 minutes). Once potential problems of understanding were resolved, the online survey was designed and a third pilot test carried out to ensure that the survey was understood and the time required for the online response remained within the estimated range. 

The following sociodemographic and professional variables were collected: age, sex, profession, years of work, participation in influenza sentinel surveillance network, and type of population (rural <10,000 and urban ≥ 10,000). We also collected the presence of risk conditions for influenza and contraindications to influenza vaccination in each worker, information on knowledge of and attitudes to influenza and vaccination, and cohabitation with children <15 years, people with chronic disease or people aged ≥ 65 years, and influenza vaccination in the 2011-2012 season and the three preceding seasons. Variables related to knowledge of and attitudes to influenza vaccination were covered by a set of questions evaluated on a Likert scale with 5 categories: totally agree, agree quite a lot, neither agree or disagree, disagree quite a lot, and totally disagree.

### Statistical analysis

The data analysis excluded workers with contraindications to vaccination and those in whom the vaccination was indicated due to risk medical conditions.

A bivariate comparison was made between vaccinated and unvaccinated workers considering the different sociodemographic variables, and professional, knowledge and attitudes using the Chi-square test. The answers to questions about knowledge and attitudes were dichotomized in two categories: positive (totally agree, agree quite a lot) and negative (neither agree or disagree, disagree quite a lot, and totally disagree). All statistical tests were two-tailed and the α error accepted was 0.05.

The trend was assessed using the χ^2^ test for linear trend.

 A multivariate analysis was performed using logistic regression with backward selection procedure of variables, with a cut-off point of <0.2.

Since vaccination in preceding seasons was the factor most strongly associated with vaccination in the study season, an analysis including these variables and another excluding them was made. 

The analysis was performed using SPSS version 18 (SPSS Inc., Chicago, IL).

### Ethics

All information collected was treated as confidential, in strict observance of legislation on observational studies. An email was sent to primary healthcare workers inviting them to participate. By clicking on the link to the questionnaire, workers implied consent to participate. As the survey was answered online, written consent was not sought. The initial email explained that all answers would be anonymous. In the stored data, respondents were identified only by a number. The study protocol, including the consent procedure, was approved by the Ethics and Clinical Research Committee of the Jordi Gol Institute for Research in Primary Care.

## Results

The questionnaire was sent to 5433 PHC workers, of whom 2635 started the questionnaire and 1965 (36.2% of those contacted) completed it.

**Figure 1:**
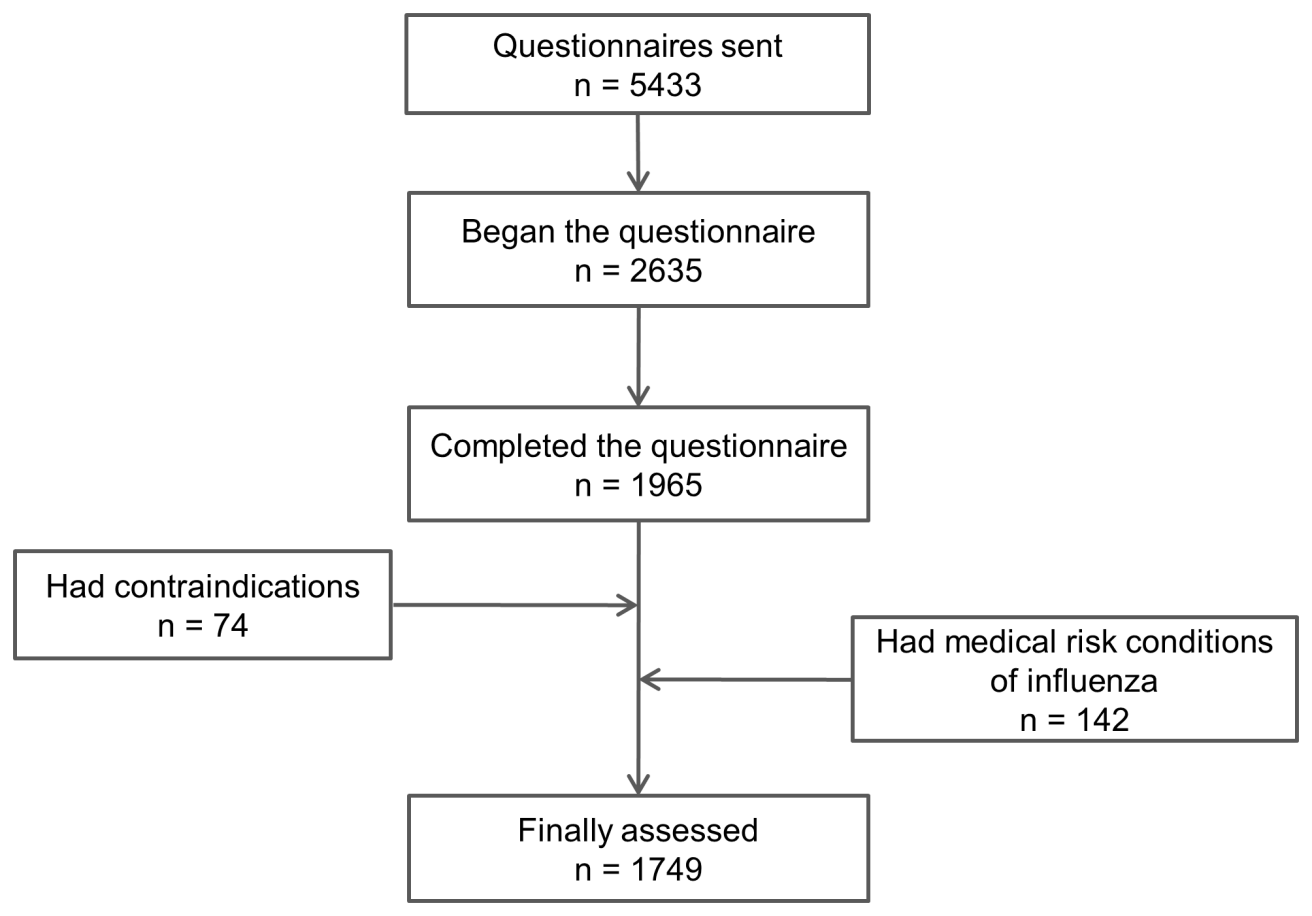
Flowchart of healthcare workers’ questionnaires assessed in the study.

Of the workers who answered the questionnaire, 74 had contraindications to influenza vaccination and 142 had ≥1 health risks for influenza and were excluded. Therefore, 1749 workers were finally analysed (Figure 1). The sociodemographic characteristics of workers initially included and those finally analysed are shown in table S1. Workers finally analysed were younger than those included in the initial sample (the proportion of subjects aged ≥55 years was 24.7% and 30.7%, respectively; p<0.01), there were fewer males (25.9 and 29.0%, respectively; p=0.03) and there were more nurses (43.1% and 37.8%, respectively, p<0.01).

The overall vaccination coverage was 50.7% and increased with age (p value of trend < 0.001) (Table 1), with the highest coverage in the ≥ 55 years age group (aOR 2.09, 95% CI 1.35-3.23). Vaccination was lower in females than in males (aOR 0.75, 95% CI 0.59 to 0.96). Coverage was higher in paediatricians than in family physicians (aOR 1.58, 95% CI 1.12-2.24). Living with a person with chronic disease (aOR 2.06, 95% CI 1.48-2.86) and living with a person aged ≥ 65 years (aOR 1.40, 95% CI 1.03-1.91) were associated with vaccination. 

**Table 1:** Distribution of vaccinated and unvaccinated healthcare workers by demographic and professional characteristics, Spain 2011-2012.

	**Vaccinated /N (%)**	**Crude OR (95% CI)**	***P* value**	**Adjusted OR^a^ (95% CI)**	***P* value**
**Age^b^**					
25-34 years	42/123 (34.1)	1		1	
35-44 years	198/440 (45)	1.58 (1.04 - 2.39)	0.03	1.52 (0.99 - 2.35)	0.06
45-54 years	405/753 (53.8)	2.24 (1.51 - 3.35)	<0.001	2.09 (1.38 - 3.17)	<0.001
≥55 years	241/433 (55.7)	2.42 (1.59 - 3.68)	<0.001	2.09 (1.35 - 3.23)	0.001
**Sex**					
Male	260/453 (57.4)	1		1	
Female	626/1296(48.3)	0.69 (0.56 - 0.86)	0.001	0.75 (0.59 - 0.96)	0.02
**Professional category**					
Family physician	421/816 (51.6)	1		1	
Paediatrician	113/179 (63.1)	1.61 (1.15 - 2.24)	0.005	1.58 (1.12 - 2.24)	0.01
Nurse	352/754 (46.7)	0.82 (0.67 - 1.00)	0.05	0.90 (0.72 - 1.12)	0.35
**Years of work**					
≤ 9 years	61/145 (42.1)	1		1	
10-29 years	577/1147 (50.3)	1.39 (0.98 - 1.97)	0.06	0.97 (0.62 - 1.53)	0.91
≥ 30 years	248/457 (54.3)	1.63 (1.12 - 2.38)	0.01	0.88 (0.51 - 1.53)	0.66
**Participant in influenza sentinel network**					
No	808/1602 (50.4)	1		1	
Yes	78/147 (53.1)	1.11 (0.79 - 1.56)	0.54	0.94 (0.66 - 1.34)	0.74
**Type of population**					
Rural	178/348 (51.1)	1		1	
Urban	682/1351 (50.5)	0.97 (0.77 - 1.23)	0.82	0.96 (0.76 - 1.23)	0.77
**Children <15yr in the household**					
No	532/1041 (51.1)	1		1	
Yes	354/708 (50.0)	0.96 (0.79 - 1.16)	0.65	1.13 (0.90 - 1.42)	0.28
**Living with persons with chronic disease**					
No	744/1536 (48.4)	1		1	
Yes	142/213 (66.7)	2.13 (1.57 - 2.88)	<0.001	2.06 (1.48 - 2.86)	<0.001
**Living with persons aged ≥65 yr**					
No	736/1509 (48.8)	1		1	
Yes	150/240 (62.5)	1.75 (1.32 - 2.32)	<0.001	1.40 (1.03 - 1.91)	0.03

^a^ Adjusted for the following variables: Age, Sex, Professional category, Cohabitation with person with chronic disease, Cohabitation with person aged ≥ 65 years

^b^ Test for linear trend: p-value < 0.001

Table 2 shows the relationship between vaccination and knowledge of and recommendations about influenza. The variables associated with vaccination were positive responses to questions about whether the worker recommended vaccination for pregnant women in their first trimester (aOR 1.45, 95% CI 1.15-1.84), in their second or third trimester (aOR 1.70, 95% CI 1.26-2.29), and postpartum (aOR 1.34, 95% CI 1.05-1.71) and whether they recommended vaccination to people aged ≥65 years (aOR 4.99, 95% CI 1.56-15.91).

**Table 2:** Influenza vaccination coverage of healthcare workers according to knowledge and attitudes on influenza vaccination, Spain 2011-2012.

	**Vaccinated/N (%)**	**Crude OR (95% CI)**	***P* value**	**Adjusted OR (95% CI)**	***P* value**
**What strain does the influenza vaccine contain?**					
A	83/156 (53.2)	1		1	
B	29/68 (42.6)	0.65 (0.37 - 1.16)	0.15	0.60 (0.33 - 1.09) ^a^	0.09
C	6/9 (66.7)	1.76 (0.42 - 7.29)	0.44	2.03 (0.47 - 8.61) ^a^	0.34
A and B	663/1300 (51)	0.91 (0.66 - 1.28)	0.60	0.93 (0.66 - 1.31) ^a^	0.69
No response	105/216 (48.6)	0.83 (0.55 - 1.26)	0.38	0.90 (0.59 - 1.37) ^a^	0.62
**What strains are responsible for epidemics?**					
A	110/216 (50.9)	1		1	
B	57/120 (47.5)	0.87 (0.56 - 1.36)	0.55	0.92 (0.58 - 1.46) ^b^	0.73
C	4/7 (57.1)	1.28 (0.28 - 5.88)	0.75	1.41 (0.30 - 6.67) ^b^	0.66
A and B	624/1206 (51.7)	1.03 (0.77 - 1.38)	0.82	1.08 (0.80 - 1.45) ^b^	0.63
No response	91/200 (45.5)	0.80 (0.55 - 1.18)	0.27	0.92 (0.61 - 1.37) ^b^	0.68
**Influenza has an incubation period of 10 -14 days**					
No	414/780(53.1)	1		1	
Yes	405/845 (47.9)	0.81 (0.67 - 0.99)	0.04	0.86 (0.71 - 1.06) ^a^	0.16
No response	67/124 (54)	1.04 (0.71 - 1.52)	0.84	1.16 (0.79 - 1.71) ^a^	0.46
**Influenza is not transmitted by contact**					
No	553/1082 (51.1)	1		1	
Yes	295/593 (49.7)	0.95 (0.77 - 1.16)	0.59	0.92 (0.75 - 1.13) ^a^	0.42
No response	38/74 (51.4)	1.01 (0.63 - 1.62)	0.97	0.96 (0.59 - 1.57) ^a^	0.89
**I recommend the vaccine to pregnant women in their first trimester**					
No	348/737 (47.2)	1		1	
Yes	289/506 (57.1)	1.49 (1.18 - 1.87)	0.001	1.45 (1.15 - 1.84) ^c^	0.002
**I recommend the vaccine to pregnant women in their second or third trimester**					
No	96/227 (42.2)	1		1	
Yes	564/1053 (53.6)	1.57 (1.18 - 2.10)	0.002	1.70 (1.26 - 2.29) ^a^	0.001
**I recommend the vaccine to post partum women**					
No	263/553 (47.6)	1		1	
Yes	322/581 (55.4)	1.37 (1.08 - 1.73)	0.01	1.34 (1.05 - 1.71) ^d^	0.02
**I recommend the vaccine to persons aged ≥ 65 years**					
No	4/19 (21.1)	1		1	
Yes	792/1570 (50.4)	3.82(1.26 - 11.55)	0.02	4.99(1.56 - 15.91) ^e^	0.007
**I recommend the vaccine to people with chronic disorders**					
No	7/16 (43.8)	1		1	
Yes	861/1691 (50.9)	1.33 (0.49 - 3.60)	0.57	1.90 (0.67 - 5.34) ^a^	0.22
**I recommend the vaccine to immunosuppressed people**					
No	47/107 (43.9)	1		1	
Yes	784/1521 (51.5)	1.36 (0.91 - 2.01)	0.13	1.38 (0.92 - 2.06) ^b^	0.12
**Any specific training in the influenza in the last five years**					
No	541/1101 (49.1)	1		1	
Yes	345/648 (53.2)	1.18 (0.97 - 1.43)	0.10	1.07 (0.87 - 1.30) ^a^	0.53

^a^ Adjusted for the following variables: Age, Sex, Professional category, Cohabitation with person with chronic disease, Cohabitation with person aged ≥ 65 years

^b^ Adjusted for the following variables: Age, Sex, Professional category, Influenza has an incubation period of 10 -14 days? Cohabitation with person with chronic disease, Cohabitation with person aged ≥ 65 years

^c^ Adjusted for the following variables: Age, Sex, Cohabitation with person with chronic disease, Cohabitation with person aged ≥ 65 years, What strain does the influenza virus contain?, What strains are responsible for epidemics?

^d^ Adjusted for the following variables: Age, Sex, Cohabitation with person with chronic disease, Cohabitation with person aged ≥ 65 years, What strain does the influenza virus contain?, What strains are responsible for epidemics? , Influenza has an incubation period of 10 -14 days?

^e^ Adjusted for the following variables: Age, Sex, Cohabitation with person with chronic disease, Cohabitation with person aged ≥ 65 years, Influenza is transmitted by respiratory aerosols

Vaccination in all three preceding seasons (aOR 9.76, 95% CI 7.18-13.28), in any of the three preceding seasons (aOR 7.63, 95% CI 4.93-11.80), and vaccination with the pandemic vaccine in 2009-2010 (aOR 2.16, 95% CI 1.60-2.93) were closely associated with vaccination in 2011-12 (table S2). The relationship between attitudes to influenza and influenza vaccination is shown in table 3. The closest association was with concern about infection at work (aOR 4.93, 95% CI 3.72-6.53), followed by considering vaccination of HCW important (aOR 2.62, 95% CI 1.83-3.75), concern about becoming ill (aOR 2.44, 95% CI 1.85-3.21), the belief that vaccination is effective in preventing influenza and its complications (aOR 2.40, 95% CI 1.56-3.67) and the belief that vaccination of high risk individuals is effective in reducing complications (aOR 2.38, 95% CI 1.16-4.86). Table S3 shows the relationships between influenza vaccination coverage and attitudes towards influenza and influenza vaccine adjusted by the vaccination history in preceding seasons. The associations between vaccination and concern about infection at work (aOR 2.90, 95% CI 2.02-4.16), considering vaccination of HCW important (aOR1.69, 95% CI 1.11-2.57), concern about becoming ill (aOR 1.73, 95% CI 1.19-2.51) and the belief that vaccination is effective in preventing influenza and its complications (aOR 2.10, 95% CI 1.24-3.53) were slightly weaker than those obtained before adjustment. In contrast, the association with the belief that vaccination of high risk individuals is effective in reducing complications (aOR 2.60, 95% CI 1.07-6.34) was slightly stronger.

**Table 3:** Influenza vaccination coverage of healthcare workers according to attitudes towards influenza and the influenza vaccine, Spain 2011-2012.

	**Vaccinated/N (%)**	**Crude OR (95% CI)**	***P* value**	**Adjusted OR (95% CI)^a^**	***P* value**
Concern about infection at work	673/878 (76.7)	10.14 (8.14 - 12.63)	<0.001	4.93 (3.72 - 6.53)	<0.001
Influenza can be a serious illness	599/1113 (53.8)	1.42 (1.16 - 1.72)	<0.001	0.77 (0.57 - 1.05)	0.09
Vaccination is effective in preventing influenza and its complications	844/1493 (56.5)	6.63 (4.69 - 9.37)	<0.001	2.40 (1.56 - 3.67)	<0.001
Concern about becoming ill	610/826 (73.8)	6.62 (5.37 - 8.16)	<0.001	2.44 (1.85 - 3.21)	<0.001
Concern about infecting patients	679/1060 (64.1)	4.15 (3.38 - 5.10)	<0.001	1.51 (1.14 - 2.02)	0.005
Vaccination of healthcare workers is important	805/1306 (61.6)	7.18 (5.51 - 9.36)	<0.001	2.62 (1.83 - 3.75)	<0.001
Vaccination of persons at high risk is effective in reducing the complications of the disease	869/1653 (52.6)	5.15 (3.02 - 8.78)	<0.001	2.38 (1.16 - 4.86)	0.01
Vaccination of healthcare workers reduces outbreaks	657/1065 (61.7)	3.20 (2.62 - 3.91)	<0.001	1.21 (0.91 - 1.62)	0.19
Vaccination is the most important measure in preventing influenza infection	818/1445 (56.6)	4.53 (3.39 - 6.05)	<0.001	1.50 (1.00 - 2.28)	0.05
Pandemic influenza caused a heavier workload than seasonal influenza	534/1022 (52.3)	1.10 (0.90 - 1.34)	0.34	0.74 (0.57 - 0.95)	0.02
Pandemic influenza had a more severe presentation than seasonal influenza	225/409 (55)	1.22 (0.97 - 1.52)	0.08	0.93 (0.70 - 1.25)	0.63
Activities carried out during 2009-10 were adjusted to the evolution of the pandemic	281/497 (56.5)	1.34 (1.09 - 1.66)	0.006	0.85 (0.65 - 1.12)	0.26

^a^ Adjusted for the following variables: Age, Sex, Professional category, Cohabitation with person with chronic disease, Cohabitation with person aged ≥ 65 years, Vaccination of healthcare workers is important, Vaccination of persons at high risk is effective in reducing complications, Vaccination of healthcare workers reduces the risk of outbreaks, Vaccination is the most important measure in preventing influenza infection , Pandemic influenza caused a heavier workload than seasonal influenza, I am concerned about catching influenza in the workplace, I think that influenza can be a severe disease, I think the influenza vaccination is effective in preventing influenza and its complications, I worry about catching influenza, I worry about giving influenza to my patients.

## Discussion

Our results show that vaccination of PHC workers is associated with concerns about influenza in the workplace, considering vaccination of healthcare workers as important, and the belief that vaccination is effective in preventing influenza and its complications and in reducing complications in high risk individuals. No association was found between vaccination and knowledge of influenza or the characteristics of the vaccine. 

The proportion of workers who responded to the questionnaire (36.2%) is similar to or even higher than that obtained by studies of vaccination coverage in hospital and PHC workers in several countries [10,24,28-30]. In Andalusia (Spain) [14] a response of 73% was found in PHC workers. Aerny et al [27], after conducting online questionnaires in various groups of healthcare workers found response rates ranging between 25% (in PHC workers) and 63%. The differences may be due to the organization and burden of work between different types of healthcare workers. 

The overall influenza vaccination coverage observed (50.7%) was lower than the 57.7% found in people aged ≥ 65 years in Spain, the group for whom vaccination is absolutely recommended, as it is for healthcare workers [31]. Likewise, an Austrian study by Blank et al found that coverage among HCW was lower than that found in people aged ≥ 65 years (15.5% and 32.1%, respectively) [25].

This coverage was close to the 58% found by Blasi et al [32] in a survey carried out among members of two European societies and to that found by Bouadna et al (58% for physicians and 30% for other healthcare workers) in a French hospital study [33]. Compared with other studies in primary healthcare carried out in Spain, the coverage was somewhat higher than the 44.2% and 19.6% obtained by Ortiz et al [14] and Jimenez et al [4], respectively. Studies in PHC physicians by Picazo et al [34] and Martinez et al [35] found rates of 75% and 88.3%, respectively. If we had not excluded workers in whom vaccination was contraindicated, the coverage would have been lower (48%). 

Vaccination coverage was highest in workers aged ≥ 55 years (55.7%), males (57.4%), paediatricians (63.1%) and workers living with a person with chronic disease (66.7%).

The results of other studies conducted in Spain that include PHC [4,14] or hospital [7,13] workers agree that vaccination coverage in healthcare workers increases with age. Studies carried out including non-hospital workers in other countries [10,17,28,36] found similar results.

Kaboli et al [28] found that vaccination coverage was higher in workers working in more urbanized areas, a result not observed in our study.

The higher vaccination coverage in males was also observed by other authors [14,17,36]. 

In agreement with the results of the study by Kaboli et al [28], we did not find that nurses had lower coverages than family physicians, although other studies have found the opposite [5-7,17,20,33,36]. Likewise, we found that paediatricians had higher coverages, confirming the results of the study by Bertin et al [37]. 

The reluctance of heath care workers to accept influenza vaccination has been associated with lack of of knowledge about influenza infection [10,20], but we found no differences between vaccinated and unvaccinated workers about the virus strain included in the vaccine, the mechanisms of transmissions and other epidemiological characteristics of the disease. Only 12.3% of study subjects did not know what influenza virus strains were included in the seasonal vaccine, much smaller than the 78.3% found by Esposito et al in Italian hospital workers [20]. Our results suggest that, although education on influenza infection and vaccine characteristics are important aspects, education is not always translated into vaccination. Interestingly, we found an association between the vaccination of workers and recommending the vaccine to pregnant women in their second or third trimester (aOR 1.70, 95% CI 1.26-2.29) or postpartum (aOR 1.34, 95% CI 1.05 -1.71, similar to the results of a Slovenian study by Socan et al which found that unvaccinated HCW were more reluctant to vaccinate pregnant women than vaccinated HCW [30]. The recommendation to vaccinate pregnant women is supported by studies showing that pregnant women develop protective levels of antibodies after vaccination [38-40], and that vaccination during pregnancy has the added benefit of providing passive transfer of influenza antibodies to neonates [41].

Our results show that 99.5% of vaccinated workers recommended vaccination to patients aged ≥ 65 years, slightly higher than 96.3% obtained in the study by Abramson et al [17]. 

As in other studies [17,28,33,36,42,43], vaccination in all or some of the preceding three seasons were the factors with the closest association with seasonal vaccination. 

Although vaccination of patients with high risk conditions has been shown to be effective in reducing complications [1], the belief that the vaccine is not effective and that influenza is not a severe disease is common [15-18]. In our study, 10% of unvaccinated workers held these beliefs, similar to the findings of Ajenjo et al [44] in a large nonprofit healthcare organization and higher than the results found by Optelsten et al [42] (4.1%) in general practitioners, but lower than the figures found in other studies with PHC workers [17,36,45]. 

Concern about influenza infection was associated with vaccination, with 76% of vaccinated respondents reporting they were concerned about influenza in the workplace, a percentage higher than that found by other authors [36,42]. Concern about becoming ill (aOR 2.44, 95%CI 1.85-3.21) and concern about infecting patients (aOR 1.51, 95% CI 1.14-2.02) were also associated with vaccination of HCW, confirming the results of a meta-analysis of Italian studies that showed that self- protection and the protection of patients were the main ideas encouraging vaccination [46].

The main strengths of this study are the number of subjects included, higher than most studies carried out in PHC workers, the high proportion of the Spanish population covered by the regions included and the exclusion of workers in whom vaccination was contraindicated or who had chronic high risk conditions. Thus, only workers who were candidates for vaccination due to their work status were included.

Self-report is a possible limitation of the study. However, the results of several studies have shown very good agreement between self-reported influenza vaccination status and medical records [47,48] and it seems unlikely [33] that this factor may have invalidated our results.

Selection bias is another possible limitation: although centres were selected randomly, we cannot know the proportion of vaccinated workers among non responders, due to the anonymous nature of the questionnaire. Differences in knowledge and attitudes between responders and non responders were not assessable as these data were not available for non responders. Respondents may have been more motivated to respond to queries about influenza and influenza vaccination, as other authors have suggested [32]. However, we compared the sociodemographic characteristics of all workers invited to participate and of those finally assessed, and found that workers who completed the questionnaire completely were younger (the proportion of subjects aged >55 years was 30.7% in the initial sample and 24.7% in those who completed the questionnaire). In contrast, the proportion of nurses was higher in workers finally assessed (43.1%) than in those initially selected (37.8%), but no differences in vaccination coverage were observed between nurses and family physicians. As the results of the study show that younger people had lower vaccination rates, the possible selection bias in this case might have resulted in an underestimate of the vaccination coverage. However, we cannot exclude the possibility that there are other unknown differences between respondents and non-respondents.

Finally, because this study included nearly 900 vaccinated workers and a similar number of unvaccinated workers, we believe we have captured a wide range of opinions and concerns about influenza and its prevention by means of vaccination and therefore the results on attitudes to influenza and influenza vaccination may reflect real differences between vaccinated and unvaccinated PHC workers in Spain.

In conclusion, our findings suggest that paediatricians and older healthcare workers are more compliant with vaccination in PHC, but that there are no important differences in knowledge of influenza and the influenza vaccine. The lack of vaccination in healthcare workers should be considered a professional error. Educational programs should aim to remove misconceptions and attitudes that limit compliance with recommendations on influenza vaccination in PHC workers rather than just increasing knowledge on influenza infection and the characteristics of the vaccine.

## Supporting Information

Table S1 **Distribution of characteristics of all healthcare workers the questionnaire was sent to and those finally analysed.**
(DOC)

Table S2 **Association between influenza vaccination coverage of healthcare workers and vaccination in preceding seasons.** Spain, 2011-2012.
(DOC)

Table S3 **Influenza vaccination coverage of healthcare workers according to attitudes towards influenza and the influenza vaccination, including influenza vaccination in preceding seasons, Spain 2011-2012.**
(DOC)

## References

[1] Fiore AC, Bridges CB, Katz JM, Cox NJ (2012) Inactivated influenza vaccines. In: Plotkin SA Orenstein WA Offit PA Vaccines. 6th ed. Philadelphia: Elsevier pp. 257-293.

[2] Centro Nacional de Epidemiología (2011) Vigilancia de casos graves hospitalizados confirmados de gripe en España. Temporada. Madrid: Instituto de Salud Carlos III pp. 2010-2011. Available: http://www.isciii.es/ISCIII/es/contenidos/fd-servicios-cientifico-tecnicos/fd-vigilancias-alertas/fd-enfermedades/Informe_casos_graves_hospitalizados_2010- _07septiembre2011.pdf. Accessed 28 June 2013

[3] Centres for Disease Control and Prevention, CDC (2013) Prevention strategies for seasonal influenza in healthcare settings. Guidelines and Recommendations. Available: http://www.cdc.gov/flu/professionals/infectioncontrol/healthcaresettings.htm. Accessed 29 June 2013

[4] Jiménez-García R, Hernández-Barrera V, Carrasco-Garrido P, Sierra-Moros MJ, Martinez-Hernandez D et al. (2006) Influenza vaccination coverages among Spanish children, adults and health care workers. Infection 34: 135-141. PubMed: 16804656. 16804656 10.1007/s15010-006-5627-1

[5] Galicia-García MD, González-Torga A, García-González C, Fuster-Pérez M, Garrigós-Gordo I et al. (2006) Influenza vaccination in healthcare workers. Why are some vaccinated whereas others are not. Enferm Infecc Microbiol Clin 24: 413-417. PubMed: 16956528. 16956528 10.1157/13091777

[6] Elorza JM, Campins M, Martínez X, Allepuz A, Ferrer E et al. (2002) Vacuna antigripal y personal sanitario: estrategias para aumentar las coberturas en un hospital de tercer nivel. Med Clin (Barc) 119: 451-452. 12385652 10.1016/s0025-7753(02)73452-0

[7] Bautista D, Vila B, Uso R, Tellez M, Zanon V (2006) Predisposing, reinforcing and enabling factors influencing influenza vaccination acceptance among healthcare workers. Infect Control Hosp Epidemiol 27: 73-77. PubMed: 16418992. 16418992 10.1086/499148

[8] Miller BL, Ahmed F, Lindley MC, Wortley PM (2011) Increases in vaccination coverage of healthcare personnel following institutional requirements for influenza vaccination: a national survey of U.S. hospitals. Vaccine 29: 9398-9403. PubMed: 21945495. 21945495 10.1016/j.vaccine.2011.09.047

[9] Vaux S, Van Cauteren D, Guthmann JP, Le Strat Y, Vaillant V et al. (2011) Influenza vaccination coverage against seasonal and pandemic influenza and their determinants in France: a cross-sectional survey. BMC Public Health 11: 30. doi:10.1186/1471-2458-11-S3-S30. PubMed: 21226919. 21226919 PMC3025842

[10] Kraut A, Graff L, McLean D (2011) Behavioral change with influenza vaccination: factors influencing increased uptake of the pandemic H1N1 versus seasonal influenza vaccine in health care personnel. Vaccine 29: 8357-8363. doi:10.1016/j.vaccine.2011.08.084. PubMed: 21888939. 21888939

[11] Arda B, Durusoy R, Yamazhan T, Sipahi OR, Taşbakan M et al. (2011) Did the pandemic have an impact on influenza vaccination attitude? A survey among health care workers. BMC Infect Dis 11: 87. doi:10.1186/1471-2334-11-87. PubMed: 21473763. 21473763 PMC3084177

[12] García de Codes A, Arrazola MP, de Juanes JR, Hernández MT, Jaén F et al. (2010) Campaña de vacunación antigripal (pandémica y estacional) en trabajadores de un hospital general (2009-2010). Vacunas 11: 49-53. doi:10.1016/S1576-9887(10)70011-X.

[13] Vírseda S, Restrepo A, Arranz E, Magán-Tapia P, Fernández-Ruiz M et al. (2010) Seasonal and pandemic A (H1N1)2009 influenza vaccination coverage and attitudes among health-care workers in a Spanish University Hospital. Vaccine 28: 4751-4757. doi:10.1016/j.vaccine.2010.04.101. PubMed: 20471438. 20471438 PMC7115598

[14] Ortiz MA, Abd KM, Caballero JM, Allam MF (2011) Coverage and side effects of influenza A (H1N1)2009 monovalent vaccine among primary health care workers. Vaccine 29: 6366-6368. doi:10.1016/j.vaccine.2011.04.117. PubMed: 21840463. 21840463

[15] Hollmeyer HG, Hayden F, Poland G, Buchholz U (2009) Influenza vaccination of health care workers in hospitals - A review of studies on attitudes and predictors. Vaccine 27: 3935-3944. doi:10.1016/j.vaccine.2009.03.056. PubMed: 19467744. 19467744

[16] Ofstead CL, Tucker SJ, Beebe TJ, Poland GA (2008) Influenza vaccination among registered nurses: information receipt, knowledge, and decision-making at an institution with a multifaceted educational program. Infect Control Hosp Epidemiol 29: 99-106. doi:10.1086/526431. PubMed: 18179363. 18179363

[17] Abramson ZH, Levi O (2008) Influenza vaccination among primary healthcare workers. Vaccine 26: 2482-2489. doi:10.1016/j.vaccine.2008.03.011. PubMed: 18407385. 18407385

[18] Maltezou HC, Maragos A, Katerelos P, Paisi A, Karageorgou K et al. (2008) Influenza vaccination acceptance among health-care workers: a nationwide survey. Vaccine 26: 1408-1410. doi:10.1016/j.vaccine.2008.01.049. PubMed: 18313179. 18313179

[19] Smedley J, Poole J, Waclawski E, Stevens A, Harrison J et al. (2007) Influenza immunization: attitudes and beliefs of UK healthcare workers. Occup Environ Med 64: 223-227. PubMed: 17182640. 17182640 10.1136/oem.2005.023564 PMC2078449

[20] Esposito S, Bosis S, Pelucchi C, Tremolati E, Sabatini C et al. (2008) Influenza vaccination among healthcare workers in a multidisciplinary university hospital in Italy. BMC Public Health 8: 422. doi:10.1186/1471-2458-8-422. PubMed: 19105838. 19105838 PMC2651144

[21] Hofmann F, Ferracin C, Marsh G, Dumas R (2006) Influenza vaccination of healthcare workers: a literature review of attitudes and beliefs. Infection 34: 142-147. doi:10.1007/s15010-006-5109-5. PubMed: 16804657. 16804657

[22] Böhmer MM, Walter D, Müters S, Krause G, Wichmann O (2011) Seasonal influenza vaccine uptake in Germany 2007/2008 and 2008/2009: results from a national health update survey. Vaccine 29: 4492-4498. doi:10.1016/j.vaccine.2011.04.039. PubMed: 21545822. 21545822

[23] Beguin C, Boland B, Ninane J (1998) Health care workers: vectors of influenza virus? Low vaccination rate among hospital health care workers. Am J Med Qual 13: 223-227. doi:10.1177/106286069801300408. PubMed: 9833335. 9833335

[24] Nichol KL, Hauge M (1997) Influenza vaccination in healthcare workers. Infect Control Hosp Epidemiol 18: 189-194. doi:10.1086/647585. PubMed: 9090547. 9090547

[25] Blank PR, Freiburghaus AU, Schwenkglenks MM, Szucs TD, Kunze U (2008) Influenza vaccination coverage rates in Austria in 2006/7 – a representative cross-sectional telephone survey. Wien Med Wochenschr 158: 583-588. doi:10.1007/s10354-008-0582-3. PubMed: 18998078. 18998078

[26] Maltezou HC, Katerelos P, Poufta S, Pavli A, Maragos A et al. (2013) Attitudes toward mandatory occupational vaccinations and vaccination coverage against vaccine-preventable diseases of health care workers in primary health care centres. Am J Infect Control 41: 66-70. doi:10.1016/j.ajic.2012.01.028. PubMed: 22709989. 22709989

[27] Aerny N, Domínguez MF, Astray J, Esteban-Vasallo MD, Blanco LM et al. (2012) Tasas de respuesta a tres estudios de opinión realizados mediante cuestionarios en línea en el ámbito sanitario. Gac Sanit 26: 477-479. doi:10.1016/j.gaceta.2011.10.016. PubMed: 22361641. 22361641

[28] Kaboli F, Astrakianakis G, Li G, Guzman J, Naus M et al. (2010) Influenza vaccination and intention to receive the pandemic H1N1 influenza vaccine among healthcare workers of British Columbia, Canada: A cross-sectional study. Infect Control Hosp Epidemiol 31: 1017-1024. doi:10.1086/655465. PubMed: 20707670. 20707670

[29] Chor JSY, Pada SK, Stephenson I, Goggins WB, Tambyah PA et al. (2011) Seasonal influenza vaccination predicts pandemic H1N1 vaccination uptake among healthcare workers in these countries. Vaccine 29: 7364-7369. doi:10.1016/j.vaccine.2011.07.079. PubMed: 21807048. 21807048

[30] Sočan M, Erčulj V, Lajovic J (2013) Knowledge and attitudes on pandemic and seasonal influenza vaccination among Slovenian physicians and dentists. Eur J Public Health 23: 92-97. doi:10.1093/eurpub/cks006. PubMed: 22366387. 22366387

[31] Ministerio de Sanidad, Servicios Sociales e Igualdad (2013). Coberturas de Vacunación. Datos estadísticos. Available at http://www.msssi.gob.es/profesionales/saludPublica/prevPromocion/vacunaciones/coberturas.htm accessed 29 June 2013

[32] Blasi F, Palange P, Rohde G, Severin T, Cornaglia G et al. (2011) Healthcare workers and influenza vaccination: an ERS-ESCMID Web-based survey. Clin Microbiol Infect 17: 1223-1225. doi:10.1111/j.1469-0691.2011.03501.x. PubMed: 21595785. 21595785

[33] Bouadma L, Barbier F, Biard L, Esposito-Farèse M, Le Corre B et al. (2012) Personal decision-marking criteria related to seasonal and pandemic A(H1N1) influenza-vaccination acceptance among french healthcare workers. PLOS_ONE 7: e38646 PubMed: 22848342. 22848342 10.1371/journal.pone.0038646 PMC3407215

[34] Picazo JJ, González F, Salleras L, Bayas JM, Alvarez MJ (2012) Survey of adult influenza and pneumococcal vaccination in Spain. Vacunas 13: 100-111. doi:10.1016/S1576-9887(12)70048-1.

[35] Martínez F, Martínez P, Sequí A, Beviá I, Ruiz M et al. (2011) Flu vaccination coverages in primary health careworkers: season 2005/6 to 2009/10. Vacunas 12: 48-51. doi:10.1016/S1576-9887(11)70005-X.

[36] LaVela SL, Smith B, Weaver FM, Legro MW, Goldstein B et al. (2004) Attitudes and practices regarding influenza vaccination among healthcare workers providing services to individual with spinal cord injuries and disorders. Infect Control Hosp Epidemiol 25: 933-940. doi:10.1086/502323. PubMed: 15566027. 15566027

[37] Bertin M, Scarpelli M, Proctor AW, Sharp J, Robitson E et al. (2007) Novel use of the intranet to document health care personnel participation in a mandatory influenza vaccination reporting program. Am J Infect Control 33: 33-37. 10.1016/j.ajic.2006.10.005 17276789

[38] Eick AA, Uyeki TM, Klimov A, Hall H, Reid R et al. (2011) Maternal influenza vaccination and effect on influenza virus infection in young infants. Arch Pediatr Adolesc Med 165: 104-111. doi:10.1001/archpediatrics.2010.192. PubMed: 20921345. 20921345

[39] Benowitz I, Esposito DB, Gracey KD, Shapiro ED, Vázquez M (2010) Influenza vaccine given to pregnant women reduces hospitalization due to influenza in their infants. Clin Infect Dis 51: 1355-1361. doi:10.1086/657309. PubMed: 21058908. 21058908 PMC3106242

[40] Mak TK, Mangtani P, Leese J, Watson JM, Pfeifer D (2008) Influenza vaccination in pregnancy: current evidence and selected national policies. Lancet Infect Dis 8: 44-52. doi:10.1016/S1473-3099(07)70311-0. PubMed: 18156088. 18156088

[41] Zaman K, Roy E, Arifeen SE, Rahman M, Raqib R et al. (2008) Effectiveness of maternal influenza immunization in mothers and infants. N Engl J Med 359: 1555-1564. doi:10.1056/NEJMoa0708630. PubMed: 18799552. 18799552

[42] Opstelten W, van Essen GA, Heijnen ML, Ballieux MJP, Goudswaard AN (2010) High vaccnation rates for seasonal and pandemic (A/H1N1) influenza among healthcare workers in Dutch general practice. Vaccine 28: 6164-6168. doi:10.1016/j.vaccine.2010.07.031. PubMed: 20659516. 20659516

[43] Looijmans-van den Akker I, Marsaoui B, Hak E, van Delden JJ (2009) Beliefs on mandatory influenza vaccination of health care workers in nursing homes: A questionaire study from the Netherlands. J Am Geriatr Soc 57: 2253-2256. doi:10.1111/j.1532-5415.2009.02560.x. PubMed: 20121988. 20121988

[44] Ajenjo MC, Babcok KF, Gemeinhart N, Jones M (2010) Influenza vaccination among healthcare workers: ten-year experience of a large healthcare organization. Infect Control Hosp Epidemiol 31: 233-240. doi:10.1086/650449. PubMed: 20055666. 20055666 PMC3919446

[45] Tanguy M, Boyeau C, Pean S, Marijon E, Delhumeau A et al. (2011) Acceptance of seasonal and pandemic A(H1N1) 2009 influenza vaccination by healthcare workers in a French teaching hospital. Vaccine 29: 4190-4194. doi:10.1016/j.vaccine.2011.03.107. PubMed: 21497636. 21497636

[46] La Torre G, Mannocci A, Ursillo P, Bontempi C, Firenze A et al. (2011) Prevalence of influenza vaccination among nurses and ancillary workers in Italy: systematic review and meta analysis. Hum Vaccin 7: 728-733. doi:10.4161/hv.7.7.15413. PubMed: 21705859. 21705859

[47] Zimmerman RK, Raymund M, Janosky JE, Nowalk MP, Fine MJ (2003) Sensitivity and specificity of patient self-report of influenza and pneumococcal polysaccharide vaccination among elderly outpatients in diverse patient care strata. Vaccine 21: 1486-1491. doi:10.1016/S0264-410X(02)00700-4. PubMed: 12615445. 12615445

[48] Loulergue P, Moulin F, Vidal-Trecan G, Absi Z, Demontpion C et al. (2009) Knowledge, attitudes and vaccination coverage of healthcare workers regarding occupational vaccinations. Vaccine 27: 4240-4243. doi:10.1016/j.vaccine.2009.03.039. PubMed: 19481314. 19481314

