# Supplementary material for: Knowledge of and Attitudes to Influenza Vaccination in Healthy Primary Healthcare Workers in Spain, 2011-2012
Source: PLoS One. 2013 Nov 18;8(11):e81200. doi: 10.1371/journal.pone.0081200 (PMC3832596; doi:10.1371/journal.pone.0081200)
Supplement: Table S1 — Distribution of characteristics of all healthcare workers the questionnaire was sent to and those finally analysed. (DOC) [file pone.0081200.s001.doc]

|  | **All healthcare workers the questionnaire was sent to  (n=5433)** | **Healthcare workers analysed (n=1749)** | ***P* value** |
| --- | --- | --- | --- |
| **Age** |  |  |  |
| <24 years | 0.1% | - | - |
| 25-34 years | 6% | 7% |  |
| 35-44 years | 20.5% | 25.2% | 0.07 |
| 45-54 years | 42.7% | 43.1% | 0.12 |
| ≥55 years | 30.7% | 24.7% | <0.01 |
| **Sex** |  |  |  |
| Male | 29% | 25.9% |  |
| Female | 71% | 74.1% | 0.03 |
| **Professional category** |  |  |  |
| Physician | 49.9% | 46.7% |  |
| Paediatrician | 12.3% | 10.2% | 0.09 |
| Nurse | 37.8% | 43.1% | <0.01 |
| **Type of population** |  |  |  |
| Rural | 21.2% | 20% |  |
| Urban | 78.8% | 80% | 0.36 |
