# Supplementary material for: Knowledge of and Attitudes to Influenza Vaccination in Healthy Primary Healthcare Workers in Spain, 2011-2012
Source: PLoS One. 2013 Nov 18;8(11):e81200. doi: 10.1371/journal.pone.0081200 (PMC3832596; doi:10.1371/journal.pone.0081200)
Supplement: Table S2 — Association between influenza vaccination coverage of healthcare workers and vaccination in preceding seasons. Spain, 2011-2012. (DOC) [file pone.0081200.s002.doc]

|  | **Vaccinated/N (%)** | **Crude OR (95% CI)** | ***P* value** | **Adjusted OR (95% CI)** | ***P* value** |
| --- | --- | --- | --- | --- | --- |
| Seasonal vaccination in any of the three preceding seasons | 854/1212 (70.5) | 37.65 (25.80 – 54.94) | <0.001 | 7.63 (4.93 – 11.80)^a^ | <0.001 |
| Seasonal vaccination in all the three preceding seasons | 710/812 (87.4) | 30.10 (23.10 – 39.21) | <0.001 | 9.76 (7.18 – 13.28)^a^ | <0.001 |
| Vaccination with pandemic vaccine in 2009-2010 | 579/736 (78.7) | 8.48 (6.80 – 10.58) | <0.001 | 2.16 (1.60 – 2.93)^b^ | <0.001 |

^a^Adjusted for the following variables: Age, Sex, Professional category, Type of population, Living with children, Cohabitation with person with chronic disease, Cohabitation with person aged ≥ 65 years, Pandemic vaccine

^b^Adjusted for the following variables: Age, Sex, Professional category, Type of population, Living with children, Cohabitation with person with chronic disease, Cohabitation with person aged ≥ 65 years, Seasonal vaccination
