# Supplementary material for: Knowledge of and Attitudes to Influenza Vaccination in Healthy Primary Healthcare Workers in Spain, 2011-2012
Source: PLoS One. 2013 Nov 18;8(11):e81200. doi: 10.1371/journal.pone.0081200 (PMC3832596; doi:10.1371/journal.pone.0081200)
Supplement: Table S3 — Influenza vaccination coverage of healthcare workers according to attitudes towards influenza and the influenza vaccination, including influenza vaccination in preceding seasons, Spain 2011-2012. (DOC) [file pone.0081200.s003.doc]

|  | **Crude OR (95% CI)** | ***P* value** | **Adjusted OR^a^ (95%CI)** | ***P* value** |
| --- | --- | --- | --- | --- |
| Vaccination with seasonal influenza vaccine in any of the 3 preceding seasons | 37.65 (25.80 – 54.94) | <0.001 | 5.40 (3.34 – 8.75) | <0.001 |
| Vaccination with seasonal influenza vaccine in all 3 preceding seasons | 30.10 (23.10 – 39.21) | <0.001 | 7.56 (5.39 – 10.58) | <0.001 |
| Vaccination with pandemic vaccine 09-10 | 8.48(6.80 – 10.58) | <0.001 | 1.88 (1.35 – 2.62) | <0.001 |
| Concern about infection at work | 10.14 (8.14 – 12.63) | <0.001 | 2.90 (2.02 – 4.16) | <0.001 |
| Influenza can be a serious illness | 1.42 (1.16 – 1.72) | <0.001 | 0.80 (0.57 – 1.11) | 0.19 |
| Vaccination is effective in preventing influenza and its complications | 6.63(4.69 – 9.37) | <0.001 | 2.10 (1.24 – 3.53) | 0.005 |
| Concern about becoming ill | 6.62 (5.37 – 8.16) | <0.001 | 1.73 (1.19 – 2.51) | 0.004 |
| Concern about infecting patients | 4.15 (3.38 – 5.10) | <0.001 | 1.87 (1.24 – 2.81) | 0.003 |
| Vaccination of healthcare workers is important | 7.18 (5.51 – 9.36) | <0.001 | 1.69 (1.11 – 2.57) | 0.02 |
| Vaccination of persons at high risk is effective in reducing complications | 5.15 (3.02 – 8.78) | <0.001 | 2.60 (1.07 – 6.34) | 0.03 |
| Vaccination of healthcare workers reduces outbreaks | 3.20 (2.62 – 3.91) | <0.001 | 1.21 (0.85 – 1.72) | 0.30 |
| Vaccination is the most important measure in preventing influenza infection | 4.53 (3.39 – 6.05) | <0.001 | 1.11 (0.67 – 1.83) | 0.68 |
| Pandemic influenza caused a heavier workload than seasonal influenza | 1.10 (0.90 – 1.34) | 0.34 | 0.77 (0.56 – 1.06) | 0.10 |
| Pandemic influenza had a more severe presentation than seasonal influenza | 1.22 (0.97 – 1.52) | 0.08 | 1.01 (0.68 – 1.42) | 0.94 |
| Activities carried out during 2009-2010 were adjusted to the evolution of the pandemic | 1.34 (1.09 – 1.66) | 0.006 | 0.88 (0.63 – 1.24) | 0.46 |

^a^ Adjusted for the following variables: Age, Sex, Professional category, Vaccination in any of the 3 preceding seasons, Vaccination in all 3 preceding seasons, Pandemic vaccine, Cohabitation with person with chronic disease, Cohabitation with person aged ≥ 65 years, Vaccination of healthcare workers is important, Vaccination of persons at high risk is effective in reducing complications, Pandemic influenza caused a heavier workload than seasonal influenza, I am concerned about catching influenza in the workplace, I think the influenza can be a serious illness, I think the influenza vaccination is effective in preventing influenza and its complications, I worry about catching influenza, I worry about giving influenza to my patients.
